# Piperlongumine mediated apoptosis in cervical cancer cells beyond docking predictions

**DOI:** 10.1016/j.toxrep.2025.102031

**Published:** 2025-04-23

**Authors:** Shama Parveen, Ana Ahtsham, Saurabh Kumar, Pratishtha Gupta, Faraz Ghous, Monisha Banerjee

**Affiliations:** a Molecular and Human Genetics Laboratory, Department of Zoology, University of Lucknow, Lucknow, Uttar Pradesh 226007, India; b Department of Chemistry, University of Lucknow, Lucknow, Uttar Pradesh 226007, India

**Keywords:** Anti-apoptotic proteins, Apoptosis, Cervical cancer, Molecular docking, Piperlongumine, Pro-apoptotic proteins

## Abstract

Cervical cancer is the 4th most prevalent cancer in women. Despite its global health impact, cervical cancer research has achieved limited success with various therapies (chemo, radio, chemo-radio, surgery, etc.). Recent decades have seen no improvement in survival rates due to cancer recurrence and long-term health concerns. These treatments show the need for a new herbal approach as alternative therapy for cancer. Piperlongumine is one of numerous perfected phytochemicals with anticancerous properties. In this study, in silico simulations demonstrate various novel properties of Piperlongumine. Molecular docking was performed between receptor and ligand by simulating molecular interactions in the pro and anti-apoptotic proteins B-cell lymphoma 2 (Bcl-2), Bcl-2-associated X protein (Bax) and Bcl-2 antagonist/killer (Bak). The docking scores of Piperlongumine with Bcl-2, Bak, and Bax are −7.02, −6.78, and −7.54 kcal/mol, respectively. Piperlongumine was also bio-tested for anticancer activities against cervical cancer cells (SiHa). Piperlongumine showed compromised cell viability, promoted apoptosis, higher nuclear condensation, reduction in mitochondrial membrane potential, increase in reactive oxygen species level, promoted cell cycle arrest, upregulation of pro-apoptotic gene whereas downregulation of anti-apoptotic genes. *In vitro* studies revealed that Piperlongumine targets apoptotic pathway in cervical cancer and could be a possible effective treatment for cervical cancer. Additional *in vivo* research is needed to explore the potential of Piperlongumine to improve treatment outcomes.

## Highlights

• PL showed good affinity with BCL2 protein (PDBID: 2F8U) having total binding energy of −6.90 kcal/mol.
• PL inhibited cell growth, cell migration and mitochondrial membrane potential.
• PL induced ROS generation, chromatin condensation and cell cycle arrest.
• PL demonstrated anticancer potential via apoptosis against cervical carcinoma.

## 1 Introduction

Cancer incidents and mortality burden is increasing worldwide. In 2024, the United States is projected to experience 2001,140 new cancer cases, resulting in 611,720 deaths [1], [2], [3]. Cervical cancer, primarily caused by persistent Human Papilloma Virus (HPV) infections, ranks as the fourth most prevalent malignancy in women, accounting for 604,127 new cases and 348,186 deaths in 2020 [4]. Treatment options include surgery, chemotherapy, radiation therapy, immunotherapy, and targeted therapies, but challenges such as cancer relapse, drug resistance, and non-targeted tissue toxicity persist [1].

Instead of using the chemicals, some natural bioactive compounds derived from medicinal plants influencing autophagy and apoptosis has been explored recently [5], [6]. Piperlongumine (PL) constituent of the fruit of *Piper longum*, inhibits the proliferation of cancer via activation of extrinsic and intrinsic pathways and initiating the imbalance between pro- apoptotic (Bax, Bid, Bad, Bic, Bak, Bok, Bcl-xs, and Hrk) and anti-apoptotic proteins (Bcl-2, Bcl-xl, Bcl-w, A1 and Mcl-1) to promote cell death [7]. The rationale for selecting PL over other medicinal plant compounds lies in its unique mechanism of action. Unlike many conventional chemotherapeutic agents or other phytochemicals, PL selectively increases oxidative stress in cancer cells, leading to mitochondrial membrane permeabilization and apoptosis induction. This occurs via multiple pathways, including the ROS-mediated apoptotic pathway, where PL disrupts redox homeostasis, leading to Bax/Bak oligomerization at the mitochondrial outer membrane (MOMP), cytochrome c release, and caspase. PL also induces cell cycle arrest (G2/M phase) and DNA damage response, overcoming HPV16-mediated resistance to apoptosis in SiHa cervical cancer cells by activating p53-independent apoptotic pathways. Bax/Bak adapt to various stress signals by altering conformation and assembling into oligomeric complexes in the mitochondrial outer membrane, where they then transform into pore-forming proteins., Bak undergoes significant shape changes to create the pores, allowing the proteins to form dimers that can cluster and perforate the membrane [8]. Bax and Bak induce mitochondrial dysfunction by releasing Cytochrome c, which triggers apoptotic pathways leading to cell death [9], [10]. Among the most well-known targets for chemo-sensitization are anti-apoptotic factors, specifically the Bcl2 that is B-cell lymphoma-2 family proteins [11]. Many investigations suggest that whether a cell survives or dies in response to apoptotic signalling depends on the relative quantities of these pro-apoptotic and anti-apoptotic proteins in the cell. Bcl2 elevation has also been linked to unfavourable prognoses. As a result, the Bcl2 transcriptional control has become a popular target for cancer therapies [12]. A promising computational technique for structure-based drug design is molecular docking. The main objective of which is predicting the ideal binding configuration and related binding affinity between a ligand (small molecule) and a target protein. In current study the binding affinity between PL and apoptotic (pro and anti) genes have been done along with cytotoxic assays, cell cycle analysis and gene expression study to examine the anticancerous potential of PL in SiHa cervical cancer (HPV 16 +ve) cell line.

## 2 Experimental section

### 2.1 Computational details

Molecular docking serves as a computational tool in drug development, that enables the identification of lead compounds through database screening. The docking score (kcal/mol) for the protein-ligand complex is produced when a desired ligand binds with protein/receptor binding sites in a particular orientation. The most efficient ligand is a complex with a low docking score (binding energy). The several mutual conformations that potentially lead to binding are commonly referred to as binding modes. In the current investigation, the process of docking was carried out with the automated docking programme AutoDock 4.2.6 version software, which integrates a powerful Lamarckian genetic search method as well as an empirical free energy function and permits torsional flexibility in the ligand [13]. To restore protein in their original geometry, several steps were taken. Firstly the, water molecule was removed and polar hydrogen atoms were added. Finally, the Kollman united atom partial charges were added. The ligand was prepared for docking by lowering its energy using the ADT script and the B3LYP/6–311 + +G(d,p) level of theory. It was then converted to PDBQT. AutoGrid was used to construct the grid maps that represented the proteins throughout the actual docking process. The grid dimensions were set to 60 Å × 60 Å × 60 Å with a spacing of 0.525 Å between grid points, ensuring coverage of residues from the enzyme’s active site and substantial portions of the surrounding surface. One grid was chosen for each type of atom in the ligand, and another was chosen for electrostatic interactions. High resolution 3D crystal structures of G-Quadruplex structure formed in Human Bcl-2 Promoter (PDBID: 2F8U) and antiparallel basket-type G-Quadruplex DNA structure formed in human (PDBID: 6ZX7) for the docking studies were downloaded from Protein Data Bank (http://www.rscb.org/pdb) [14], [15], [16]. The finest docked poses were visualized utilizing the PyMOL visualizer and their interactions was visualized by LigPlot+ .

### 2.2 In vitro assessments

#### 2.2.1 Cell culture

Human cervical cancer cells (SiHa), reported to have integrated copies of HPV-16 strain, were grown and maintained in Dulbecco’s Modified Eagle Medium (DMEM) supplemented with 10 % heat-inactivated Fetal Bovine Serum (FBS) and 1 % antibiotic-antimycotic solution until reaching 70–85 % confluency. The cells were trypsinized by using 0.25 % trypsin (Gibco, ThermoScietific) after removing DMEM medium from cell culture flask and incubated for 5 minutes in 5 % (v/v) CO_2_ for 5 min to detach the cells. After detachment of cells the activity of trypsin was neutralised by adding the culture medium. Collected cells were then centrifuged for 5 min at 1000 rpm and re-suspended in culture media and counted using the Trypan Blue exclusion method to assess cell viability and determine accurate seeding density for further experimental study.

#### 2.2.2 Cytotoxicity assessment

In 96 well plate, SiHa cells were seeded at a rate of 1 × 10^4^ cells in each well and incubated for 24 h. Subsequently, the cells were treated with seven concentrations of PL ranging from 5 to 35 μM (5 μM, 10 μM, 15 μM, 20 μM, 25 μM, 30 μM and 35 μM), and kept in incubation at 37ºC with 5 % CO_2_ for 24 h. MTT tetrazolium salt (Himedia, Pennsylvania, USA) was utilised to assess the cell viability of both treated and non-treated cells after 24 h. Following the incubation, a volume of 10 µl (5 mg/mL) solution of 3-(4,5-dimethylthiazol-2-yl)-2,5-diphenyltetrazolium bromide (MTT) was introduced into each well and subsequently incubated at a temperature of 37ºC for 3 h. The culture medium was discarded, and 100 µL of DMSO was added to each well to dissolve the formazan crystals. The absorbance was measured at a wavelength of 540 nm using a microplate reader (BioTek, USA), and the percentage of cells that were alive was determined [17]. Statistical analysis was conducted using the GraphPad Prism software. The formula to calculate the cell viability is-

***%Cell viability= [(control absorbance) – (test absorbance)/(control absorbance)] X 100***

#### 2.2.3 AO/PI cell viability assessment

Seeding of SiHa cells (1 ×10^5^ cells/well) into 12-wells plate was done and incubated for overnight. SiHa cells treated with two concentrations (10 µM and 20 µM) of PL for 24 h, to check the cell viability. For each sample, three randomly selected microscopic fields were used to count the number of viable and membrane-compromised/dead cells. SiHa cells were subjected to dual staining with Acridine Orange (AO) and Propidium Iodide (PI). The cells were rinsed with 1XPBS and 300 μL of an AO and 100 μL PI solution (each in a 3:1 ratio) subsequent to removing the treatment medium. Cells were then reared at room temperature for a duration of 15 minutes. After removing the dye from the cells and rinsing them with 1X PBS, the samples were examined at 200X magnification using fluorescence microscopy (Nikon Eclipse Ti2, Japan) [18].

#### 2.2.4 Nuclear condensation assessment

The condensation of nucleus was assessed using DAPI (4′,6-diamidino-2-phenylindole) fluorescent stain both in control and treated cells. SiHa cells were seeded in 12 wells plate (1 ×10^5^ cells/well) and incubated for overnight. The SiHa cells were subjected to 2 different concentrations (10 μM and 20 μM) of PL, for 24 h. After removing the culture medium, the cells were rinsed with 1XPBS and stained with DAPI (1 μg/mL) for 15–20 min. There after the cells were rinsed twice with 1XPBS to eliminate the over staining. The morphology of cellular nuclei was examined using fluorescence microscopy (Nikon Eclipse Ti2, Japan) at 200X magnification [19]. The fluorescence intensity quantified in the cells using *ImageJ*.

#### 2.2.5 Assessment of Mitochondrial membrane potential (MMP)

The MMP was assessed using the Mitotracker CMX Ros red dye. Cells were grown in 12 wells plate (1X10^5^ cells/well) and grown until it reaches its full confluency. The cells were treated with 10 and 20 μM PL for 24 h. Following that, the cells undergone a 1X phosphate-buffered saline (PBS) wash and were subsequently fixed using 4 % paraformaldehyde. After removing the fixative, the cells were rinsed three times with 1X PBS. Finally, a fluorescent microscope (Nikon Eclipse Ti2, Japan) was used to capture images at 200X magnification [19]. The fluorescence intensity was quantified in the cells using *ImageJ*.

#### 2.2.6 Measurement of reactive oxygen species (ROS)

DCFDA (2’,7’-dichlorofluorescin diacetate) was used to measure the levels of intracellular ROS. Cells were maintained in freshly prepared culture medium, in 12 wells plate (1X10^5^ cells/well) and after 24 h of incubation period, cells were treated with 2 different concentrations of PL (10 μM and 20 μM) for 24 h. Thereafter the culture media was discarded and cells were rinsed with 1X PBS twice. In well 40 μL of DCFDA dye (10 μM) was added and incubated in 37ºC for 20–30 minutes. The cells were rinsed with 1X PBS twice and were inspected under a fluorescent microscope (Nikon Eclipse Ti2, Japan) at 200X magnification [19]. The fluorescence intensity was quantified in the cells using *ImageJ*.

#### 2.2.7 Cell cycle analysis

SiHa cells were seeded in 6 well plate (1 ×10^6^ cells in each well), following a 24 h growth period from seeding to confluence (70–80 %), cells were treated with PL (10 and 20 µM). After carefully washing the treated cells twice in 1XPBS. Trypsin was used to harvest the cells from the incubation period and suspend them in new culture medium. The cells were centrifuged for five minutes at 1000 rpm, and the pellet was then suspended in 1 mL of 70 % ethanol to fix the cells. The suspension was left at −20°C for 3 h. Further, the cells were centrifuged for five minutes at 1500 rpm. For 40 minutes, the pellet was incubated at room temperature (RT) suspended in 500 µL of PI solution (50 µL/mL PI + 0.1 mg/mL Rnase-A + 0.05 % Triton X-100). The suspension was mixed with 3 mL of PBS, and it was centrifuged for 5 minutes at 1500 rpm. To prevent cell loss, the supernatant was carefully removed. After that, the pellet was suspended in 500 µL PBS and subjected to cell cycle analysis ( BD Bioscience, USA) to detect cell cycle arrest. The Cell Quest programme were used for this analysis [20].

#### 2.2.8 RNA isolation and real-time polymerase chain reaction

The extraction of total RNA was conducted from the SiHa cell line using RNA-zol®RT (Ohio, USA). The RNA was subsequently assessed and measured using the NanoDrop instrument (Wilmington, DE, USA). The cDNA synthesis kit from ThermoFisher Scientific USA was utilised for the purpose of cDNA synthesis. The RT-PCR analysis of both control and treated cells was performed in 96-well PCR plates using the DyNAmo flash SYBR Green RT-PCR kit (ThermoFisher Scientific, MA, USA) and gene-specific primers for the target genes. The Table 1 displays the primers used for the identified genes Bcl-2, Bax, and Bak. GAPDH was concurrently amplified as an endogenous control to evaluate gene expression relative to it. The ΔΔct method was employed to compute the fold changes [18].

**Table 1:** Forward and reverse primer sequences of target genes.

**Genes**	**Primers (5’-3’)**	**Tm (**^**o**^**C)**	**Amplicon (bp)**
**GAPDH**	F-CCCCACACACATGCACTTACC R- CCTAGTCCCAGGGCTTTGATT	58	156
**Bak**	F- ACGACATCAACCGACGCTAT R- ATGCCACTCTCAAACAGGCT	60	121
**Bcl−2**	F-GGGAATCGATCTGGAAATCCTC R-CCCATCAATCTTCAGCACTCT	60	115
**Bax**	F-TTGCTTCAGGGTTTCATCCA R-ACACTCGCTCAGCTTCTTG	62	98

#### 2.2.9 Statistical analysis

The experiments were conducted at least three times. Statistical significance was considered using ANOVA and Tukey’s multiple comparison test with P < 0.05 considered as significant. Values have been shown as mean ± SEM.

## 3 Results

### 3.1 Molecular docking

**Fig. 1:**
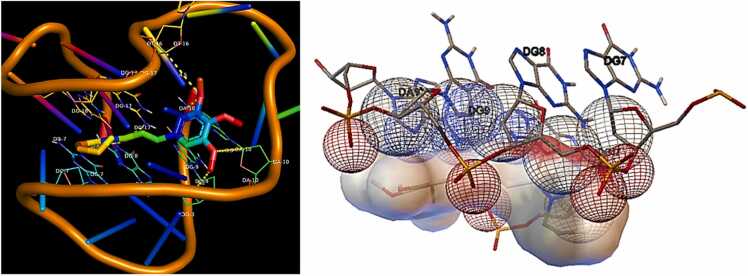
3D representation showing the interactions of Hydrogen bonding of piperlongumine with different amino acid residues at active site of Bcl-2 protein **(PDBID: 2F8U).**

According to the molecular docking studies, 2F8U reacted with different amino acids electrostatically, by Van der Waal interactions and hydrogen bonding. Two hydrogen bonds were found with several amino acid residues at binding site of 2F8U. The carbonyl oxygen atom (O_2_) formed hydrogen bonding N6···O2 (2.99 Å) and N2···O1 (2.93 Å) with amino acid residues DA 10(A) and DA 17(A) respectively. PL showed good affinity with **Bcl-2 protein (PDBID: 2F8U)** Fig. 1 having total binding energy of**-6.90 kcal/mol**(Table 2).

**Table 2:** Details of the molecular docking data of Bcl-2 proteins, showing the summary of binding energies (kcal/mol) and the H-bond as hydrophobic interactions.

**PDBID**	**Residues involved** **in Hydrogen** **Bond** **interactions**	**Residues involved in** **Hydrophobic interaction**	**No. of Bonds**		**Inhibition Constant** **Ki** **(uM)**	**Binding Energy (ΔG)** **(kcal/mol)**	
			**Number** **Hydrogen** **Bonds**	**Number of** **of Hydrophobic** **Bonds**			
**2F8U**	DA 10(A) N6……O2 **2.99 (Å)** DA 17(A) N2……O1 **2.93 (Å)**	DG 7(A), DG 8(A), DG 9(A), DG 10(A), DT 16(A), DG17(A).	2	6	8.77		**−6.90**
**6ZX7**	DC 7(A) O3’……O **3.19 (Å)**	DC 7(A), DG 8(A), DG 10(A), DG 22(A), DG 23(A), DG 24(A), DA 25(A).	1	7	7.18		**−7.02**

**Fig. 2:**
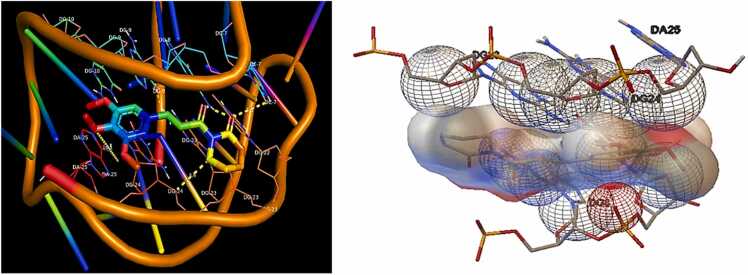
3D representation showing the interactions of Hydrogen bonding of PL with different amino acid residues at active site of Bcl-2 protein **(PDBID: 6ZX7).**

**Fig. 3:**
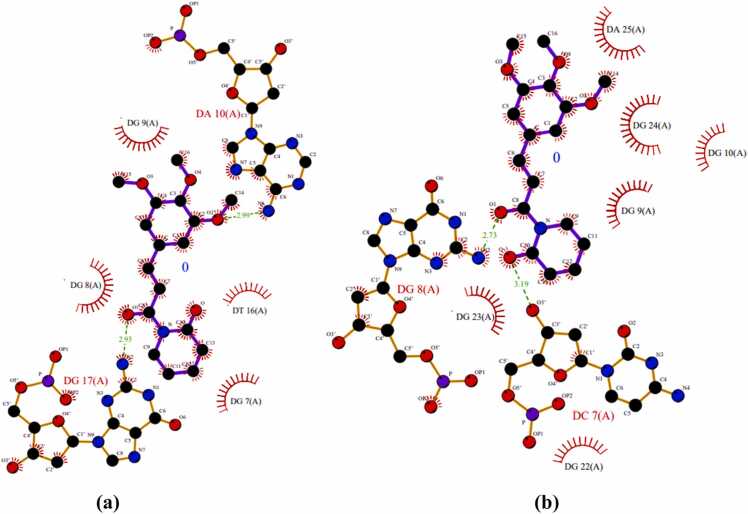
2D representation showing interactions of Hydrogen bonding in piperlongumine with different amino acid residues at active site of with Bcl-2 proteins **(a) 2F8U, (b) 6ZX7.**

One hydrogen bond was generated with the amino acid residue at binding site of 2F8U. The oxygen atom of carbonyl (O) formed hydrogen bond O3’···O (3.19 Å) with amino acid residue DC 7(A). Good binding affinity was showed by PL with Bcl-2 protein (PDBID: 6ZX7) (Fig. 2) with the total binding energy of **-7.02 kcal/mol**. Fig. 3 represents hydrogen bonds interactions presents between different amino acid residues of protein.

**Fig. 4:**
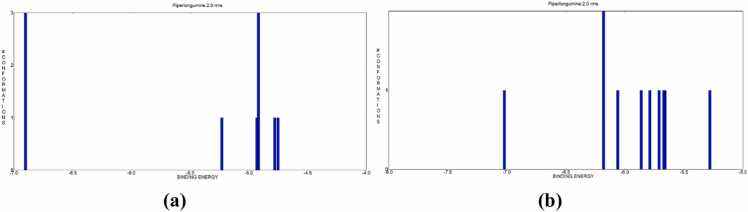
Histograms of PL with Bcl2 proteins **(a) 2F8U, (b) 6ZX7** with binding energies out of 20 runs using a RMSD-tolerance of 2.0 Å with different conformational clusters.

The optimal docked conformations were identified by analysing the clustering histogram at various RMSD values. These values quantify the Root Mean Square Deviation (RMSD) between AutoDock4’s predicted conformation and the original structure, focusing on low binding energy conformations (Table 3). The conformations in all the bins of the clustering histogram fall within 2 RMSD of the ideal docking configuration. Compound clustering histograms with the Bcl-2 protein shown in (Fig. 4). The calculated binding energies for piperlongumine and the Bcl-2 protein are shown in (Table 4).

**Table 3:** Cluster analysis of confirmations with PL RMSD values and binding energies of Bcl-2 proteins **(PDBID: 2F8U and 6ZX7).**

**Bcl-2 (PDBID: 2F8U)**				**Bcl-2 (PDBID: 6ZX7)**			
Cluster Rank	Confirmation Score	Binding Energy (kcal/mol)	RMSD (Å)	Cluster Rank	Confirmation Score	Binding Energy (kcal/mol)	RMSD (Å)
1	7	−6.90	0.00	**1**	10	−7.02	0.00
2	10	−5.23	0.00	**2**	6	−6.18	0.00
3	2	−4.93	0.00	**3**	4	−6.06	0.00
4	5	−4.92	0.00	**4**	7	−5.86	0.00
5	9	−4.78	0.00	**5**	3	−5.79	0.00
6	4	−4.75	0.00	**6**	5	−5.71	0.00
				**7**	2	−5.67	0.00
				**8**	1	−5.66	0.00
				**9**	9	−5.28	0.00

**Table 4:** Estimated binding energies for PL with Bcl-2 proteins.

**PDBID**	Estimated Free Energy of Binding (kcal/mol)	Final Intermolecular Energy (kcal/mol)	vdW + Hbond + desolv Energy (kcal/mol)	Electrostatic Energy (kcal/mol)	Final Total Internal Energy (kcal/mol)	Torsional Free Energy (kcal/mol)	Unbound System’s Energy [= (2)] (kcal/mol)
**Bcl−2 (PDBID: 2F8U)**	−6.90	−8.39	−7.64	−0.75	−0.75	+ 1.49	−0.75
**Bcl−2 (PDBID: 6ZX7)**	−7.02	−8.51	−8.20	−0.31	−0.76	+ 1.49	−0.76

**Fig. 5:**
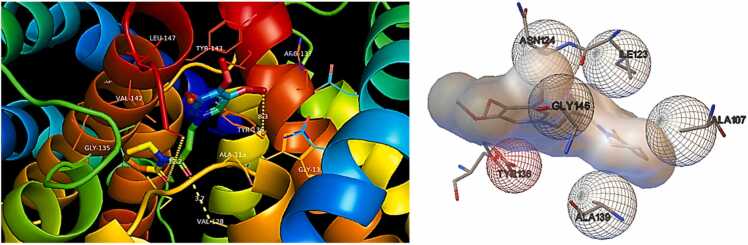
3D representation of Hydrophobic bonding interactions of PL with different amino acid residues at active site of **Bak protein (PDBID: 6ODH).**

According to the molecular docking studies with Bak protein **(PDBID: 6ODH)**, 6ODH reacted with different amino acids electrostatically, by Van der Waal interactions bonding. No hydrogen bonds and 14 hydrophobic intersections with several amino acid residues at binding site of 6ODH. Piperlongumine showed good affinity with **Bak protein (PDBID: 6ODH)**(Fig. 5) having total binding energy of −6.78 kcal/mol (Table 5).

**Table 5:** The molecular docking data including information on binding energies (in kcal/mol) and the presence of hydrogen bonds and hydrophobic interactions for **Bak (PDBID: 6ODH)** and **Bax (PDBID: 1YZ1)** proteins.

**PDBID**	**Residues involved** **in Hydrogen** **Bond** **interactions**	**Residues involved in** **Hydrophobic interaction**	**No. of Bonds**		**Inhibition Constant** **Ki** **(uM)**	**Binding Energy (ΔG)** **(kcal/mol)**
			**Number** **Hydrogen** **Bonds**	**Number of** **of Hydrophobic** **Bonds**		
**Bak (PDBID: 6ODH)**	None	Asn 86(B), Tyr 143©, Gly 146©, Val 142©, Ala 107©, Ala 139©, Leu 138©, Tyr 136(B), Met 123(A), Val 128(A), Asn 124(A), Trp 125(A), Asp 90(B), Arg 137(B).	0	14	10.66	**−6.78**
**Bax (PDBID: 1YZ1)**	None	Thr 84(A) Gln 133(D), Glu 80(D), Lys 130(A), Tyr 132(D), Tyr 132(A), Lys 130(D), Asn 131(A), Gln 133(A), Phe 129(D), Glu 80(A), Thr 84(D), Lys 85(D), Phe 83(A), Ser 82(D), Phe 143(D), Thr 81(D).	0	17	3.00	**−7.54**

**Fig. 6:**
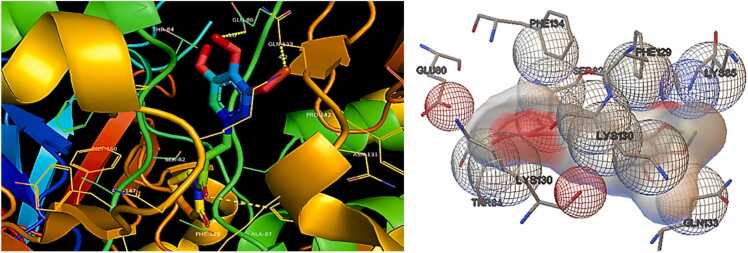
3D representation of hydrophobic bonding interactions of PL with different amino acid residues at active site of **Bax protein (PDBID: 1YZ1).**

17 hydrophobic bonds were generated with the amino acid residue at binding site of **Bax protein (PDBID: 1YZ1).** Good binding affinity was showed by piperlongumine with Bax protein Fig. 6 with the total binding energy of −7.54 kcal/mol. Fig. 3 represents hydrophobic bonds presents between different amino acid residues of protein.

**Fig. 7:**
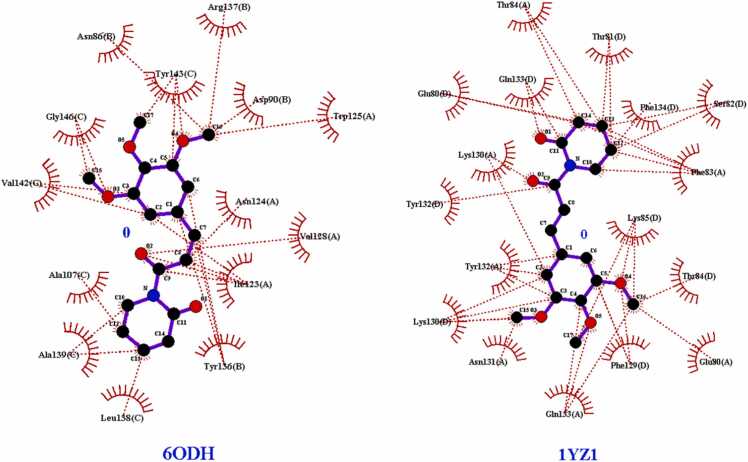
2D presentation in PL showing hydrogen bond interactions with different amino acid residues at active site of **Bak (PDBID: 6ODH)** and **Bax (PDBID: 1YZ1)** proteins.

Hydrophobic bonds present between different amino acid residues of protein are shown in Fig. 7.

**Fig. 8:**
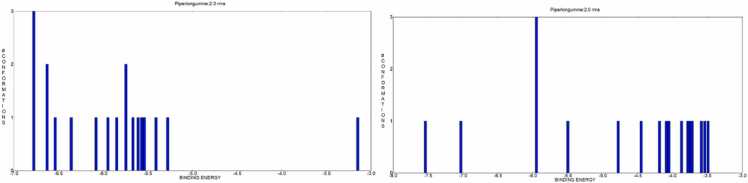
Histograms of PL with **Bak (PDBID: 6ODH)** and **Bax (PDBID: 1YZ1)** proteins having binding energies out of 20 runs using a RMSD-tolerance of 2.0 Å with different conformational clusters.

The best docked conformations were selected using the clustering histogram with different RMSD values, which determines the RMSD (Root Mean Square Deviation) between AutoDock4's predicted conformation and the actual structure, focusing on low binding energy conformations. (Table 6). Within each bin of the clustering histogram conformations that are within 2 RMSD of the ideal docking conformation were found. Compound clustering histograms using Bak and Bax protein are displayed in Fig. 8. The calculated binding energies for piperlongumine with the Bak and Bax protein are shown in (Table 7).

**Table 6:** Cluster analysis of confirmations with PL RMSD values and binding energies of **Bak (PDBID: 6ODH)** and **Bax (PDBID: 1YZ1)** proteins.

**Bak (PDBID: 6ODH)**				**Bax (PDBID: 1YZ1)**			
**Cluster Rank**	**Confirmation Score**	**Binding Energy (kcal/mol)**	**RMSD (Å)**	**Cluster Rank**	**Confirmation Score**	**Binding Energy (kcal/mol)**	**RMSD (Å)**
1	9	−6.78	0.00	**1**	7	−7.54	0.00
2	17	−6.63	0.00	**2**	14	−7.03	0.00
3	7	−6.54	0.00	**3**	12	−5.95	0.00
4	5	−6.36	0.00	**4**	19	−5.50	0.00
5	13	−6.08	0.00	**5**	18	−4.78	0.00
6	19	−6.08	0.00	**6**	20	−4.45	0.00
7	14	−5.95	0.00	**7**	15	−4.19	0.00
8	4	−5.85	0.00	**8**	6	−4.09	0.00
9	20	−5.75	0.00	**9**	10	−4.05	0.00
10	10	−5.67	0.00	**10**	4	−3.87	0.00
11	3	−5.61	0.00	**11**	5	−3.78	0.00
12	11	−5.57	0.00	**12**	11	−3.75	0.00
13	2	−5.54	0.00	**13**	16	−3.74	0.00
14	12	−5.41	0.00	**14**	8	−3.72	0.00
15	16	−5.28	0.00	**15**	2	−3.72	0.00
16	15	−3.15	0.00	**16**	13	−3.59	0.00
				**17**	9	−3.54	0.00
				**18**	3	−3.49	0.00

**Table 7:** Estimated binding energies for PL with **Bak (PDBID: 6ODH)** and **Bax (PDBID: 1YZ1)** proteins.

PDBID	Estimated Free Energy of Binding (kcal/mol)	Final Intermolecular Energy (kcal/mol)	vdW + Hbond + desolv Energy (kcal/mol)	Electrostatic Energy (kcal/mol)	Final Total Internal Energy (kcal/mol)	Torsional Free Energy (kcal/mol)	Unbound System’s Energy [= (2)] (kcal/mol)
**Bak (PDBID: 6ODH)**	−6.78	−8.27	−8.22	−0.06	−0.81	+ 1.49	−0.81
**Bax (PDBID: 1YZ1)**	−7.54	−9.03	−8.76	−0.27	−0.79	+ 1.49	−0.79

### 3.2 Evaluation of cytotoxic assays

#### 3.2.1 MTT assay

**Fig. 9:**
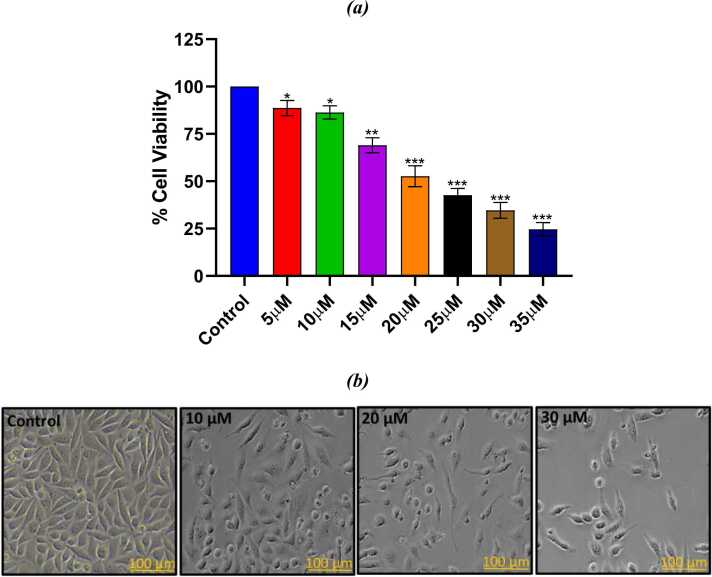
(a) The percentage of viable cells following treatment with various PL concentrations. (b) Images documented with phase contrast microscopy indicating morphological alterations in cells exposed to three distinct PL doses.

In SiHa cell PL shows dose dependent growth inhibition after 24 h of treatment as indicated by the results (Fig. 9a). The half maximum inhibitory concentration (IC_**50**_) of PL for SiHa cells was found to be 20 μM after 24 h of incubation period. Based on MTT assay, the doses for PL were selected as 10 and 20 μM for further experiments. Morphological analysis further supported the MTT assay results, as increasing concentrations of PL led to cell shrinkage, membrane blebbing and detachment from the surface, indicating apoptosis-induced cellular damage (Fig. 9b).

#### 3.2.2 Reduced cell viability

**Fig. 10:**
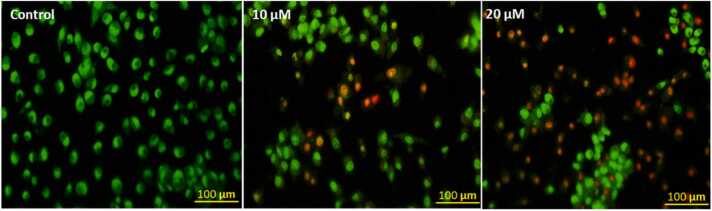
Fluorescent analysis using AO/PI staining in treated and non-treated SiHa cells.

The dual stained SiHa cells were visualized under a fluorescence microscope. Living cells with an undamaged membrane displayed green fluorescence, while damaged cells exhibited yellow-orange fluorescence and dead cells showed red fluorescence (Fig. 10). This result shows that, in comparison to the corresponding controls, the number of dead cells increased dramatically with increasing dosages of PL treatment. These findings suggested that PL promotes death of cervical carcinoma cells.

#### 3.2.3 Assessment of nuclear morphology

**Fig. 11:**
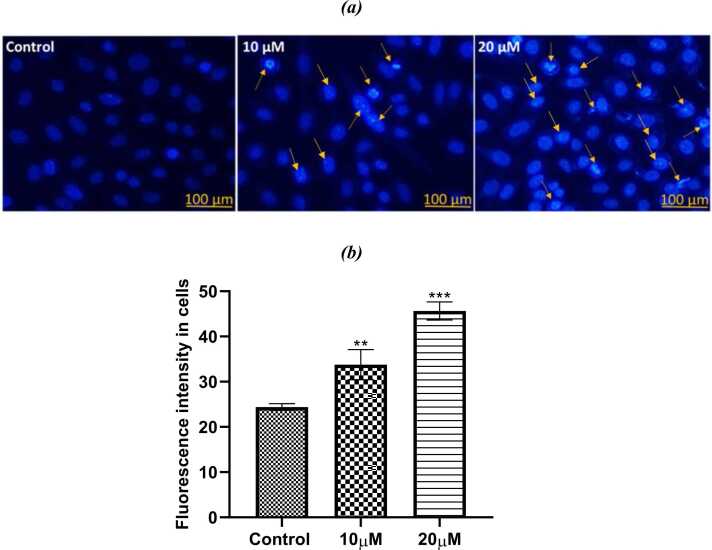
(a) The fluorescent images of nuclear condensation assessment by DAPI indicating increased apoptotic nuclei and condensation at higher concentration of PL as indicated by yellow arrows. (b) Bar diagram showing the data of three individually performed experiments in a quantified manner as Mean+SE (statistical significance, **P < 0.01, ***P < 0.001).

Fluorescence microscopy was used to analyse the changes in nucleus morphology following PL treatment. PL was found to be capable of inducing morphological alterations, chromatin condensation, and nuclear fragmentation pointed out with yellow arrows in SiHa cells, as demonstrated in (Fig. 11a, b). In addition, our findings indicate that the non-repairable nuclear damages can cause cell death through the process of apoptosis, which implicates the mechanisms of action of PL by increasing apoptosis in human cervical cancer cells, as has been observed in other cancer cells.

#### 3.2.4 Reduction in mitochondrial membrane potential

**Fig. 12:**
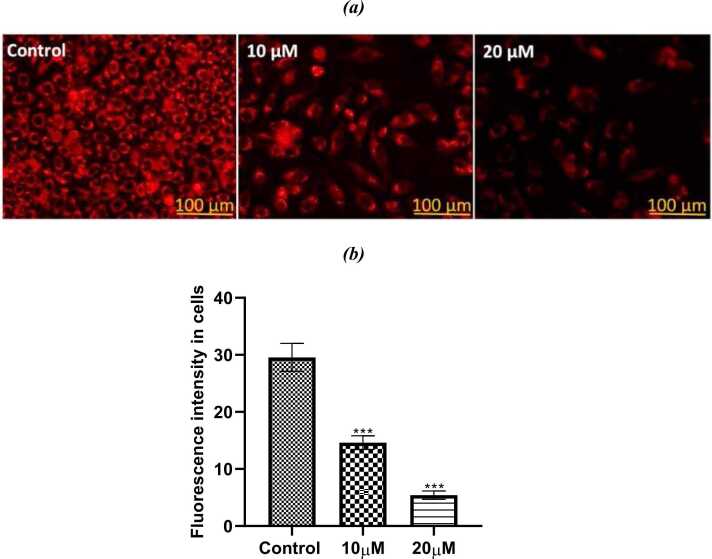
Visualization of mitochondrial membrane potential by CMXRos showing quantified data in three different set of experiments shown as Mean+SE (statical significance, ***P < 0.001).

It was found that after 24 h of different concentrations of PL, there was a notable reduction in the mitochondrial membrane potential, depending on the concentration. In the control group, the mitochondria were strongly polarized and bright red in florescence. On the other hand, the treated groups had a significant percentage of mitochondria that were poorly polarized and dim in florescence intensity. Reduced in mitochondrial potential also indicates the induction of apoptosis(Fig. 12a,b).

#### 3.2.5 Assessment of reactive oxygen species

**Fig. 13:**
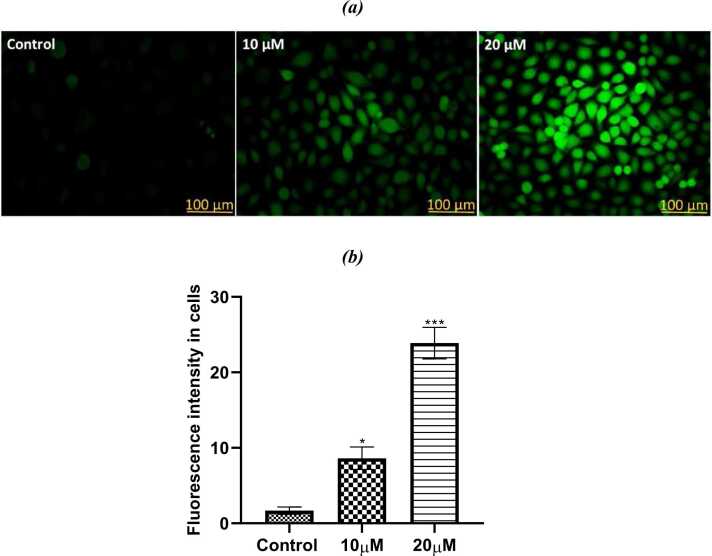
a. The fluorescence images showing the amounts of ROS in SiHa cells that were analyzed by DCFDA with two different concentrations of PL. (b) The quantified data of three individually done experiments shown as Mean+SE (statical significance, *P < 0.05, ****P < 0.0001).

Using the DCFDA fluorescent dye, the range of reactive oxygen species (ROS) inside the cells in both control and PL-treated cells were investigated. The fluorescence intensity was greatly raised in PL treated groups in comparison to the untreated controls, in which the fluorescence of DCFDA was almost nonexistent. There was a significant difference in the amounts of ROS produced by the SiHa cells when the highest dose of PL utilized in this investigation was 20 μM (Fig. 13a, b). This difference may be attributed that PL has the ability to raise the amounts of reactive oxygen species (ROS) within cells to a level that is high enough to induce a deadly oxidative stress in cells, which in turn makes cancer cells susceptible to apoptosis.

#### 3.2.6 Cell cycle arrest

**Fig. 14:**
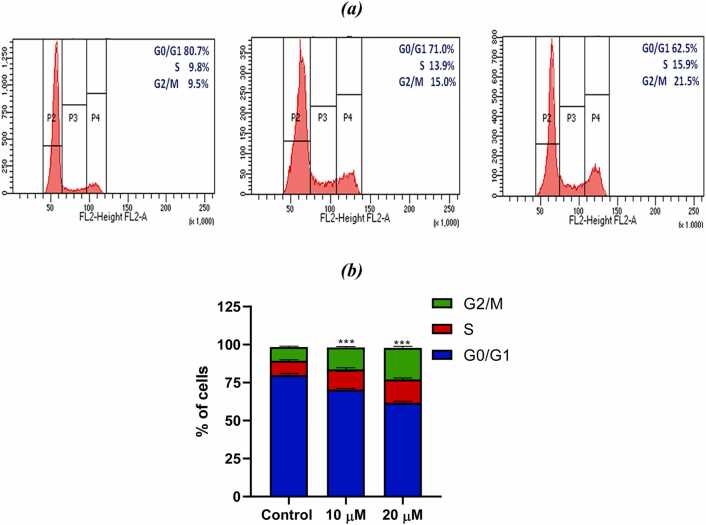
(a) Effect of PL on Cell cycle kinetics. (b) The Graph represents phase distribution of cells treated with 10 μM and 20 μM of PL.

Flow cytometry assay was done to examine the role of the PL-induced modifications on the cell cycle distribution. According to the findings, PL led to an upsurge in the proportion of cells in the G2/M phase, whereas, a minor reduction in the proportion of cells in the S phase. PL at a concentration of 20 µM was administered to SiHa cells for a period of 24 h, showed an upsurge in the percentage of cells in the G2/M phase from 6.23 % to 19.43 %. as depicted in Fig. 14b. The findings of this study suggested that PL induced cell cycle arrest by targeting apoptotic pathway and the prevention of cervical cancer cells from progressing to the G2/M phase of the cell cycle.

#### 3.2.7 Effect of PL on expression of Bcl-2, Bax and Bak genes

**Fig. 15:**
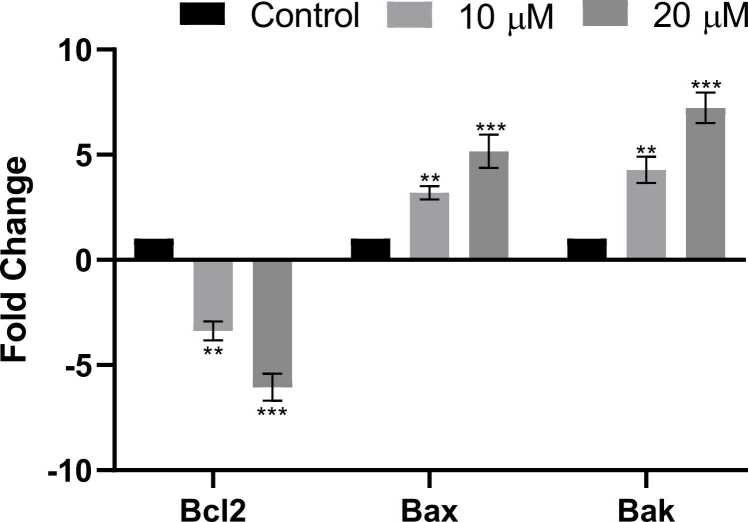
Real-time PCR showing significantly induced expression levels of the Bcl-2, Bax and Bak genes in PL treated cells. Significance is ascribed as **P < 0.01 and ***P < 0.001 as compared to control.

The expression of Bcl-2, Bax, and Bak was assessed using quantitative PCR (qPCR) to determine if it was changed following treatment with two distinct doses of PL. The qPCR analysis showed a reduced expression of Bcl-2, whereas the expression level of Bax and Bak was elevated in the PL treated groups as compared to the non-treated group (control group). (Fig. 15).

## 4 Discussion

Cancer remains a global health challenge. Cervical cancer is the most common gynaecological malignancy [21]. Treatment typically involves chemotherapy, radiation therapy, or a combination thereof, tailored to the stage of cancer. However, these treatments can have significant drawbacks, such as limited solubility, drug resistance, and damage to nearby healthy tissues [22]. To mitigate these issues, safer alternatives using plant-derived compounds are being explored. One such compound is PL, a natural alkaloid found in long pepper, which has shown promising anti-cancer properties against cervical cancer cells [23]. Our study employs molecular docking to identify potential targets of PL related to cervical carcinoma, having total binding energy of −6.90 kcal/mol. In the current study we have evaluated interaction of PL with the Bcl-2, Bax, and Bak proteins. The BCL2 family is essential in controlling apoptosis. It includes anti-apoptotic members like Bcl-2 and pro-apoptotic members like Bax and Bak [24]. Anti-apoptotic proteins have four Bcl-2 homology domains, while pro-apoptotic proteins are split into multidomain members with three domains and BH3-only molecules [11], [25], [26]. Cells lacking Bax and Bak are immune to apoptosis. These proteins are inactive in healthy cells but oligomerize and disrupt the mitochondrial membrane to trigger apoptosis in response to death signals [27]. Bcl-2 exhibits mitochondrial localization and functions as a molecular barrier to prevent proapoptotic BCL2 family members, including Bax and Bak, from permeating mitochondrial membranes [28]. This interaction leads to the release of caspase-activating death messengers into the cytosol, thereby prompting apoptosis. Molecular docking was applied in this study to predict PL’s interaction with key apoptotic regulators, particularly Bcl-2, Bax, and Bak. The docking results indicated that PL binds strongly to Bcl-2 (-7.02 kcal/mol), suggesting a potential inhibitory effect, while also interacting with Bax (-7.54 kcal/mol) and Bak (-6.78 kcal/mol), supporting its pro-apoptotic role. These computational predictions were experimentally validated, as PL treatment led to Bcl-2 downregulation and Bax/Bak upregulation in SiHa cells. Additionally, functional assays demonstrated mitochondrial membrane disruption, increased ROS production, and G2/M cell cycle arrest, aligning with the expected impact of PL’s interaction with apoptotic regulators. Thus, molecular docking provided a structural basis for PL’s mechanism of action, reinforcing its potential as an apoptosis-inducing agent in cervical cancer cells. The Oligomerization-mediated pore formation by pro-apoptotic Bax, a member of BCL2 family, is adequate to initiate mitochondrial outer membrane permeabilization (MOMP), which is subsequently accompanied by the release of apoptogenic factors including cytochrome c into the cytosol [29]. Following its binding to Aapf1, cytochrome c stimulates the formation of the apoptosome, a platform for activating caspases [30]. The balance between Bax and Bcl-2 levels is crucial for apoptosis regulation, with increased Bax promoting cell death. Bak, another apoptotic effector, facilitates apoptosis by counteracting the anti-apoptotic functions of Bcl-2 and Bcl-xl [11]. Our findings indicate that PL exhibits cancer cells death and antiproliferative effects (Fig. 10), induces nuclear condensation (Fig. 11), decreases mitochondrial membrane potential (Fig. 12), triggers reactive oxygen species (ROS) production (Fig. 13), and causes cell cycle arrest at the G2/M phase (Fig. 14) upregulation of Bax and Bak along with the down regulation of Bcl2 (Fig. 15). Mitochondrial dysfunction is a known contributor to cervical carcinoma, and apoptosis, or programmed cell death, is intricately linked to mitochondrial health through intrinsic pathways [31], [32]**.** Our research also observed that PL induces G2/M cell cycle arrest, likely due to its DNA-damaging effects. Furthermore, studies suggest that Cdk/cyclin complexes play a role in determining cell fate, with cyclin A/Cdk complexes particularly influencing apoptosis. Cdk2 activation, which follows increased expression Bax or decreased expression Bcl-2, is necessary for cell death. Additionally, Bcl-2 can impede the cell cycle's progression from G0/G1 to S phase by modulating mitochondrial metabolism. In summary, our study sheds light on the potential of PL as a complementary agent in cervical cancer treatment, offering a more targeted approach with reduced side effects.

## 5 Conclusion

The study emphasizes the significance of PL as a potential therapeutic agent in cervical cancer treatment. Interaction of PL with the BCL2 family proteins Bcl-2, Bax, and Bak suggests a mechanism by which PL can effectively induce apoptosis in cancerous cells. The anti-apoptotic protein Bcl-2 and the pro-apoptotic proteins Bax and Bak are central to the regulation of the apoptotic pathways. Ability of PL to disrupt the mitochondrial membrane potential and trigger the release of cytochrome-c, that is an apoptogenic factor into the cytosol highlights its role in promoting apoptosis through the intrinsic pathway. The activation of Cdk2, which follows the expression of Bax or repression of Bcl-2, is necessary for apoptosis to occur. Additionally, ability of Bcl-2 to modulate mitochondrial metabolism and impede the cell cycle’s progression from G0/G1 to S phase provides insight into the multifaceted role of the BCL2 family in cancer cell survival. By causing cell cycle arrest at the G2/M phase, PL may exert DNA-damaging effects that further contribute to its anti-tumor efficacy. In light of these findings, the study positions PL as a promising complementary agent in cervical cancer therapy. Its targeted approach could lead to more effective treatments with reduced side effects, offering a new avenue for patients who may not respond well to conventional therapies. The research to the existing evidence supporting the utilization of plant-derived compounds in cancer therapy, emphasizing the importance of further investigation of action mechanisms and therapeutic potential of PL.

## CRediT authorship contribution statement

**Banerjee Monisha:** Writing – review & editing, Supervision, Resources, Funding acquisition. **Gupta Pratishtha:** Methodology, Formal analysis. **Ghous Faraz:** Software, Formal analysis, Data curation. **Kumar Saurabh:** Visualization, Software, Investigation, Formal analysis. **Ahtsham Ana:** Writing – original draft, Methodology, Investigation, Formal analysis, Data curation. **Parveen Shama:** Writing – original draft, Methodology, Investigation, Formal analysis, Conceptualization.

## Declaration of Competing Interest

The authors declare that they have no known competing financial interests or personal relationships that could have appeared to influence the work reported in this paper.

## Data Availability

Data will be made available on request.
